# Self-Reported Health and Fluid Intelligence in Older Adults

**DOI:** 10.1093/geroni/igaf122.2909

**Published:** 2025-12-31

**Authors:** Liu Chen, David Salat, Steven Arnold, Hiroko Dodge

**Affiliations:** Massachusetts General Hospital, Harvard Medical School, Boston, Massachusetts, United States; Massachusetts General Hospital, Boston, Massachusetts, United States; Massachusetts General Hospital, Boston, Massachusetts, United States; Massachusetts General Hospital, Harvard Medical School, Boston, Massachusetts, United States

## Abstract

**Introduction:**

Stress, arising from physical, social, or emotional factors, can induce lasting neurobiological alterations that may contribute to cognitive impairment, particularly in domains such as fluid intelligence and memory. We hypothesized that poorer self-reported health scores, indicative of higher stress burden, would be associated with lower cognitive performance in older adults.

**Method:**

Participants’ well-being in physical, mental, and social health domains were quantified using PROMIS® Global Health measures, where higher scores indicate better health within the corresponding domain. General linear regression was then used to examine the association between health domains and cognitive factors (Memory, Crystallized Intelligence [CrystIQ], and Fluid Intelligence [FluidIQ], with higher scores indicating better cognition), while controlling for demographic variables (i.e., age, gender, race, and education). The analysis was conducted on participants aged 60 and older from the Aging Adult Brain Connectome-Human Connnectome Project - Aging (AABC-HCA) baseline visit data.

**Results:**

Health scores showed significant positive associations with FluidIQ (Physical health: coefficient=0.026 [95% CI: 0.002-0.047], P = 0.030; Mental health: coefficient=0.018 [95% CI: 0.003-0.032], P = 0.017; and Social health: 0.226 [95% CI: 0.087-0.365], P = 0.001), with the strongest associations shown in social health. No significant associations were observed for health scores and the other two cognitive factors; CrystIQ and Memory.

**Conclusion:**

Our findings demonstrate a significant link between self-reported health and FluidIQ in older adults. These findings encourage further investigation into the mechanisms linking psychosocial stress to cognitive trajectories in aging.

